# Advances in the application of tumor vaccines and combination strategies: new perspectives in lung cancer treatment

**DOI:** 10.3389/fimmu.2025.1707183

**Published:** 2026-01-21

**Authors:** Lei Sun, Qi Zhao, Liyun Miao

**Affiliations:** 1 Department of Pharmacy, Yancheng Branch of Nanjing Drum Tower Hospital, Yancheng, Jiangsu, China; 2 Department of Respiratory and Critical Care Medicine, Nanjing Drum Tower Hospital, Nanjing, Jiangsu, China

**Keywords:** combined therapy, DC vaccines, immune checkpoint inhibitors, lung cancer, tumor vaccines

## Abstract

Lung cancer remains one of the most prevalent and lethal malignancies globally and its treatment has consistently been a focal point of research in the medical field. The emergence of immunotherapies such as immune checkpoint inhibitors has brought about a new understanding of vaccine treatment for tumors. Tumor vaccines induce anti-tumor immune responses by targeting tumor-associated antigens or specific neoantigens. In recent years, advancements in vaccine technology, such as neoantigen screening, refinement of vector systems, and optimization of adjuvants, have significantly propelled the development of personalized tumor vaccines, thereby endowing lung cancer vaccines with substantial therapeutic potential. Furthermore, studies have demonstrated that the integration of tumor vaccines with immune checkpoint inhibitors, chemotherapy, and other therapeutic modalities can produce synergistic anti-tumor effects. This article reviews the latest progress in lung cancer vaccines, elucidates current combination treatment strategies supported by preclinical and clinical evidence, and explores their translational potential for clinical application.

## 1 Introduction

Lung cancer remains one of the most prevalent malignancies globally and a leading cause of cancer-related deaths (1). The primary pathological subtypes are small cell lung cancer (SCLC) and non-small cell lung cancer (NSCLC), with NSCLC accounting for approximately 85% of cases, including adenocarcinoma, squamous cell carcinoma, and large cell carcinoma (2). Lung cancer is highly aggressive, often progressing rapidly and imposing a substantial global health burden (3). Although the current treatments include chemotherapy, radiotherapy, immunotherapy, targeted therapy and other means, the long-term survival of lung cancer is still not satisfactory, and the development of effective new therapies remains an urgent need in lung cancer treatment.

Although immunotherapy represented by immune checkpoint inhibitors (ICIs) has made breakthroughs, it still faces challenges such as limited overall response rates and primary or secondary drug resistance. which may be attributed to tumor heterogeneity and the complex alterations within the tumor microenvironment (TME) (4–7). Tumor therapeutic vaccines, as an active immunotherapy, aim to activate the patient’s own immune system to specifically recognize and attack tumor cells, providing a new strategy for the precise and long-term control of lung cancer (8–10).

Tumor vaccines utilize tumor-associated antigens (TAA) or tumor-specific antigens (TSA) as immunotherapy targets to activate anti-tumor immunity and induce tumor-specific T-cell immune responses (11). TAA are self-antigens abnormally expressed by tumor cells. Studies have found that due to the existence of central and peripheral tolerance mechanisms, TAAs may not be sufficient to cause an immune response (12). In contrast, TSA, also known as neoantigens, are produced by the mutation of cancer cells, are tumor specific and often patient specific. Therefore, TSA recognized by high-affinity T cells is less likely to be affected by central tolerance and induce autoimmunity (12, 13). This characteristic enables the immune response to target more precisely, and is expected to tailor more effective and personalized treatment plans for each patient (14, 15). It is reasonable to speculate that this individualized neoantigen vaccine may be a new direction for future tumor immunotherapy (16).

In recent years, advancements in bioinformatics, genomics, and novel vaccine technologies (e.g., mRNA vaccines, dendritic cell vaccine, nanoparticle vaccines) have facilitated the design and preclinical/clinical validation of personalized cancer vaccines and support the feasibility, safety, and immunogenicity of vaccines in lung cancer treatment (17–20). However, the efficacy of single vaccine therapy is often limited. In combination with existing standard therapies, reshaping the tumor microenvironment (TME) through a synergistic mechanism is the key to maximizing its potential. tumor vaccines combination therapies exhibit significant potential. The synergistic use of vaccines with other therapy, particularly immunotherapy (such as ICIs), has been shown to enhance anti-tumor efficacy and may further improve clinical outcomes for lung cancer (21–24).

The focus on immunotherapy and the concept of precision medicine have driven the development of personalized neoantigen vaccines for lung cancer. Personalized and combined treatments represent the future direction of lung cancer therapy and tumor vaccine development. Although many new studies and clinical trials have emerged, there is a lack of comprehensive reviews on the latest progress in therapeutic vaccines for lung cancer and the combined strategies of vaccines with existing therapies, such as immune checkpoint inhibitors and targeted therapies. This article aims to summarize the latest research progress in tumor vaccines for lung cancer and deeply analyze their great potential when combined with other treatment methods. The goal is to provide a more comprehensive perspective and a clear transformation direction for future research and development.

## 2 Types and characteristics of tumor vaccines

### 2.1 Classification by antigenic targets

According to the classification of target antigens, tumor vaccines can be broadly categorized into TAA-targeted vaccines and TSA-targeted vaccines. TSA-targeted vaccines are commonly referred to as neoantigen vaccines. TAA-targeted vaccines utilize antigens that are overexpressed in tumor cells but are also present at low levels in certain normal tissues (e.g., MAGE-A3 and NY-ESO-1) (25–27). Although these vaccines can be developed as “off-the-shelf” therapeutics at relatively low cost, they are prone to immune tolerance due to their self-antigen nature, which may lead to limited immunogenicity and potential off-target effects. As a result, research focus has progressively shifted toward neoantigen vaccines, which exhibit higher immunogenicity owing to their absence in normal tissues (13). Neoantigen vaccines can be further classified into shared neoantigen vaccines and personalized neoantigen vaccines (28). Shared neoantigen vaccines target recurrent mutations observed across multiple patients (e.g., KRAS mutations), whereas personalized neoantigen vaccines are tailored to the unique mutational profile of individual patients. These vaccine types and representative candidates are summarized in **Table 1**.

**Table 1:** Types and characteristics of tumor vaccines (classified by antigenic targets).

Target type	Characteristics	Subtype	Representative products
TAA	Antigens are overexpressed on tumor cells but also expressed at low levels in some normal tissues. Autoimmune tolerance Off-target risk		MAGE-A3, NY-ESO-1, HER2, MUC1, BNT116 (BioNTech)
TSA	Antigens are tumor specific, which don’t exist in normal tissues. Strong immunogenicity High specificity and safety: No self-tolerance, extremely low risk of off-target.	Shared neoantigen vaccines	ELI-002 (Elicio) mRNA-5671 (Moderna)
		Personalized neoantigen vaccines	mRNA-4157/V940 (Moderna) GNOS-PV02 (Geneos)

TAA, tumor-associated antigen; TSA, tumor-specific antigen.

### 2.2 Classification by the delivery carrier platforms

According to delivery platforms, lung cancer vaccines are primarily categorized into the following types: cell-based vaccines (including tumor cell vaccines and dendritic cell vaccines), nucleic acid-based vaccines (such as mRNA and DNA vaccines), viral vector vaccines, and peptide or protein-based vaccines. Dendritic cells (DCs), as professional antigen-presenting cells, play a central role in initiating and regulating T cell-mediated immune responses, making DC-based vaccines a prominent focus in tumor vaccine research (17). Nucleic acid-based vaccines, particularly mRNA vaccines, function by delivering mRNA encoding specific tumor antigens into host cells, where the antigens are then expressed endogenously, enabling robust induction of cellular immunity (29). These vaccines also offer advantages in rapid design and scalable production. Peptide or protein-based vaccines deliver antigenic fragments or full-length proteins directly; although they are straightforward in design, their immunogenicity is generally limited, necessitating co-administration with potent adjuvants to enhance immune responses (30). Viral vectors have demonstrated strong oncolytic activity across various tumor models and can serve as effective carriers for tumor antigen delivery (31). Furthermore, emerging evidence suggests that certain bacterial strains, such as Escherichia coli, may also be engineered as potential vectors for cancer vaccine development (32). In recent years, advances in high-throughput sequencing technologies and immunobiology have significantly accelerated the maturation of vaccine delivery platforms. Notably, dendritic cell vaccines and mRNA vaccine technologies have achieved substantial progress and have become key areas of investigation. Given the diversity of available platforms, researchers have undertaken extensive explorations in developing novel lung cancer vaccines. The following chapter will provide a comprehensive overview of the current landscape and developmental status of various lung cancer vaccines under investigation.

## 3 The current research status of different types of tumor vaccines in lung cancer

### 3.1 Cell-based vaccines

#### 3.1.1 Tumor cell vaccines

Tumor cell vaccines represent a significant early endeavor in the development of lung cancer immunotherapy, exemplified by GVAX. GVAX is an autologous tumor vaccine genetically modified to express granulocyte-macrophage colony-stimulating factor (GM-CSF) (33, 34). Studies have demonstrated that GM-CSF gene-modified tumor vaccines can elicit tumor-specific immune responses, characterized by enhanced infiltration of T cells and dendritic cells. In patients with advanced non-small cell lung cancer (NSCLC), GVAX has shown modest antitumor activity, with an objective response rate of 18% (34). However, its clinical efficacy as a monotherapy remains limited. Consequently, current research is focused on evaluating GVAX as part of combination strategies, particularly in conjunction with immune checkpoint inhibitors.

Luis E. Raez et al. developed a vaccine based on allogeneic tumor cell lines (AD100, derived from lung adenocarcinoma) and genetically engineered these cells to express two key immunomodulatory components: B7.1 (CD80), a critical T-cell co-stimulatory molecule, and HLA-A1 or HLA-A2, major histocompatibility complex (MHC) class I molecules that facilitate the presentation of tumor antigen peptides to CD8^+^ T cells (35). This early-phase study demonstrated that the allogeneic lung adenocarcinoma cell vaccine expressing B7.1 and HLA-A molecules was well tolerated in patients with advanced NSCLC and capable of inducing robust and durable tumor-specific CD8^+^ T cell responses (35). Although the clinical response rate was low, the observed survival outcomes in this cohort—characterized by a poor baseline prognosis—were encouraging, providing valuable foundational evidence and supporting further development of cancer vaccine strategies for lung cancer.

These studies mark a pivotal shift in the conceptual understanding of lung cancer immunotherapy—from the earlier belief that “lung cancer is not amenable to immunotherapy” to the recognition that “anti-lung cancer immunity can be effectively activated through engineered vaccines.” This represents a critical milestone in the evolution of therapeutic lung cancer vaccines. The core strategy—genetically equipping tumor cells with essential immune-activating signals—has laid the groundwork for more sophisticated cellular vaccine designs and rational combinations with other immunotherapeutic modalities.

#### 3.1.2 Autologous dendritic cell vaccines

Ding, Z. and colleagues conducted the first clinical study to evaluate a personalized neoantigen peptide–pulsed autologous dendritic cell vaccine (Neo-DCVac) for the treatment of lung cancer in a single-arm, two-center trial (ChiCTR-ONC-16009100, NCT02956551) (17). The study enrolled patients with metastatic lung cancer who had undergone extensive prior therapies. Candidate neoantigens were identified through whole-exome sequencing, RNA sequencing, and bioinformatics analysis of fresh biopsy specimens. A total of 12 patients were included, receiving 85 vaccine administrations overall, with a median of 5 doses per patient (range: 3–14). Each patient received between 12 and 30 peptide-based neoantigens. All treatment-related adverse events were classified as grade 1–2, with no treatment delays attributable to toxicity. Among these patients with advanced lung cancer, the objective response rate was 25%, and the disease control rate (DCR) reached 75%. The median progression-free survival was 5.5 months, and the median overall survival was 7.9 months. This study provides the first clinical evidence supporting the use of neoantigen-pulsed DC vaccines in the treatment of advanced non-small cell lung cancer (NSCLC).

Shang et al. developed an autologous DC-based nanovaccination strategy utilizing cationic nanoparticles (cNPs) loaded with patient-derived organoids or cancer cell lysates to deliver immunogenic DC-derived microvesicles (termed cNPorganoid@MVhDC or cNPcancer cell@MVDC) (36). The use of organoids ensures a consistent and representative supply of tumor lysate while preserving key tumor-specific characteristics. In orthotopic models of pancreatic and lung cancer, this approach effectively transformed immunologically “cold” tumors into “hot” tumors, increased migratory DC infiltration, activated both T cells and natural killer (NK) cells, suppressed tumor growth, and significantly improved survival in animal models—outperforming conventional DC-based delivery methods. Mechanistically, cNPs promote the accumulation of mitochondrial DNA, thereby enhancing cGAS-STING pathway–mediated DC activation and migration. This strategy enhances antitumor immunity by reshaping the tumor microenvironment, offering a promising platform for personalized cancer immunotherapy.

Preclinical studies have demonstrated that *in situ* vaccination (ISV) using gene-modified bone marrow–derived DCs secreting C-C motif chemokine ligand 21 (CCL21) can overcome resistance to immunotherapy and induce systemic tumor-specific immune responses in murine NSCLC models (37). Immunophenotyping revealed that CCL21-DC vaccination eliminated tumor-promoting neutrophils, promoted sustained infiltration of CD8+ cytotoxic and CD4+ Th1 lymphocytes, and enriched progenitor-exhausted T cells within the tumor microenvironment (37). Currently, the CCL21-DC vaccine is undergoing evaluation in a Phase I clinical trial. However, due to variable proportions of passenger lymphocytes among individuals, the cellular composition of the CCL21-DC vaccine remains heterogeneous (38). Ingels et al. reported results from another Phase I trial assessing a patient-specific neoantigen-loaded DC vaccine in individuals with resected NSCLC (19). Systemic T cell responses were observed in 5 out of 6 vaccinated patients, with detectable T cell activity persisting up to 19 months post-vaccination. These findings indicate that individualized DC vaccines can elicit durable T cell responses in patients with advanced lung cancer.

#### 3.1.3 Allogeneic dendritic cells

Hannani, D. et al. developed a novel therapeutic vaccine based on an allogeneic plasmacytoid dendritic cell line (PDC*line), demonstrating that PDC*line cells effectively activate circulating antitumor CD8+ T cells and induce broad antitumor immune responses in lung cancer patients (39).

LI, Q etc. induced neoantigen-specific immune responses *in vivo* through dendritic cell vaccination, conveniently generating adoptive cell transfer (ACT)-adapted neoantigen-reactive T cells (NRT) (40). It was found in the pre-immunized mouse model of lung cancer that the adoptive metastasis of such NRT improved the efficacy of ACT and induced tumor regression. A research strategy was proposed for the preparation of neoantigen-reactive T cells (NRT) for ACT treatment after immunization with dendritic cell (DC) vaccines carrying oxidative tumor lysates. Their preclinical studies have shown that this DC vaccine triggers a neoantigen-specific immune response and can effectively generate NRT by pulsing DC with immunogenic neoantigens and culturing them with lymphocytes from vaccinated mice. When these NRT were adopted and transferred to mice that had been immunized with the vaccine and carried LL/2 tumors, they would launch more effective anti-tumor attacks. Furthermore, they found that the infused NRT would attach to the tumor microenvironment, and the vaccine-NRT combination would reshape this environment to prevent tumor progression. This research provides a new and convenient method for preparing NRT of ACT. The clinical transformation of this method may improve the therapeutic effect of ACT.

### 3.2 mRNA vaccines

The principle of mRNA cancer vaccines is delivery of RNA fragments encoding TAA or TSA to antigen-presenting cells (APCs), and synthesize specific antigenic proteins through the protein synthesis system of human cells. Ma, S et al. constructed an mPLA/mRNA tumor vaccine (mLPR) encoding tumor specific antigen MAGE-A1 (18). In the lung cancer mouse model, after nasal administration, the mLPR vaccine stimulates the maturation of dendritic cells, reprograms M2 macrophages into M1 macrophages, and cross-activates innate and adaptive immune responses. The mLPR vaccine inhibits the development of lung cancer and reduces bone metastasis through immune cell activation, IFN-γ/IL-12 cytokine secretion and antibody-dependent cytotoxicity mediated by natural killer cells. Nguyen, c.m. et al. evaluated the safety and therapeutic efficacy of two vaccines (synthetic long peptide and mRNA-based vaccines) designed for the same neoantigen targeting the tumor burden of Lewis lung cancer (LLC) (41). This study was conducted in the LLC homologous mouse model, and the results indicated that the mRNA-based vaccine was significantly superior to the peptide-based vaccine in preventing tumor growth in mice with low tumor burden. These results highlight the potential of mRNA vaccines as a more effective approach to treating invasive tumors and provide valuable insights for the future development of mRNA vaccines.

BNT116 is an mRNA vaccine targeting shared antigens in non-small cell lung cancer (NSCLC). An ongoing Phase I/II trial (NCT05142189) initially shows good safety and immune activation signals. The reported results showed that BNT116 Combined with the PD-1 inhibitor cemiplimab in PD-L1-positive advanced NSCLC patients (who had previously received ≤2 lines of therapy), ORR was 10%, DCR was 80%, and median PFS was 5.5months (42). The primary completion (Estimated) of this trial is 2030-02.

Moderna mrNA-4157 (V940) is an mRNA vaccine targeting personalized neoantigens. It has demonstrated therapeutic benefits and safety in clinical studies (43, 44). The phase 1 (KEYNOTE-603) study assessed the safety, tolerability, and preliminary clinical activity of mRNA-4157. The study reported that mRNA-4157 induced consistent *de novo* in patients with resected NSCLC and strengthened preexisting T-cell responses to targeted neoantigens (43). KEYNOTE-942 study reported that mRNA-4157 plus pembrolizumab prolonged recurrence-free survival versus pembrolizumab monotherapy in patients with resected high-risk melanoma and showed a manageable safety profile (44). As for safety, pyrexia, fatigue, and injection-site reactions were the most common adverse reactions with mRNA-4157. The safety profile of mRNA-4157 in combination with pembrolizumab was also largely consistent with that of pembrolizumab monotherapy (43).

### 3.3 Peptide/protein based-vaccines

Peptide/protein vaccines are the most commonly used form of tumor vaccines (45, 46). The accelerated development of new peptide drugs, including peptide drug complexes, new peptide vaccines, may drive the arrival of an era of precisely customized disease treatment plans.

Ines Mota. et al. found that ALK peptide vaccination restores the immunogenicity of ALK-rearranged non-small cell lung cancer. They identified human ALK peptides displayed by HLA-A*02:01 and HLA-B*07:02 molecules. These peptides were immunogenic in HLA-transgenic mice and were recognized by CD8 T cells from individuals with NSCLC, which paved the way for the clinical development of an ALK vaccine (47). Universal cancer peptide–based vaccine (UCPVax) is a vaccine composed of two highly selected helper peptides to induce CD4+ T helper-1 response directed against telomerase. The results of a phase Ib/IIa trial (NCT02818426) demonstrated that UCPVax was highly immunogenic and safe and provide promising 1-year OS rate (34.1%) in heavily pretreated advanced NSCLC (48). Currently, A Phase I/II clinical trial (NCT05950139) aimed to evaluate the safety of a cancer peptide vaccine to prevent or delay acquired resistance in advanced ALK+ lung cancer patients is currently recruiting.

The CIMAvax-EGF vaccine is a recombinant protein-based therapeutic vaccine composed of human epidermal growth factor (EGF) chemically conjugated to the P64K protein derived from Neisseria meningitidis serogroup B. Produced through recombinant biotechnology platforms such as bacterial or yeast expression systems, the vaccine is designed to induce the host immune system to generate polyclonal antibodies against EGF (49). By targeting circulating EGF in the bloodstream, the vaccine aims to neutralize this growth factor and thereby interrupt signaling pathways that promote tumor cell proliferation, resulting in indirect tumor inhibition The vaccine has completed systematic clinical development and validation in Cuba. A randomized, controlled Phase III clinical trial demonstrated that CIMAvax-EGF, when administered as a “switch maintenance therapy” following first-line platinum-based chemotherapy, significantly prolonged overall survival in patients with advanced non-small cell lung cancer (NSCLC). Among patients who received at least four doses of the induction regimen, median overall survival was 12.43 months, compared to 9.43 months in the best supportive care group, with a favorable safety profile. Adverse events were predominantly mild to moderate, including grade 1–2 injection site reactions and transient fever (49). Notably, subgroup analysis revealed that patients with high baseline serum EGF levels (>870 pg/mL) derived greater survival benefit, suggesting that serum EGF concentration may serve as a predictive biomarker for treatment response (49). Subsequent real-world evidence has further corroborated its efficacy and safety in routine clinical practice. A study conducted at the National Cancer Institute of Cuba reported that, under real-world conditions, patients receiving maintenance vaccination achieved a median overall survival of 14.6 months and a median progression-free survival of 8.16 months (50). Importantly, a large-scale Phase IV implementation study demonstrated the successful decentralization of vaccine administration—from tertiary hospitals to community-level polyclinics—via primary care physicians. In a cohort of 741 patients with advanced NSCLC, the vaccine maintained an excellent safety profile in this community setting, with less than 1% incidence of serious treatment-related adverse events, and provided a median survival of 12 months among patients with stable disease after first-line chemotherapy (51).

BEC2/BCG is an anti-idiotypic antibody vaccine that targets the highly expressed ganglioside antigen GD3 in tumors such as SCLC and melanoma (52). BEC2 is a monoclonal antibody whose unique structure mimics the epitope of the GD3 antigen. Therefore, after being injected as an “internal imaging” vaccine, it aims to induce the body to produce antibodies that can recognize and attack GD3-expressing tumor cells (Ab3 response) (53). However, a Phase III randomized trial of patients with limited-stage small cell lung cancer who achieved remission after chemotherapy showed that the vaccine failed to improve the overall survival or progression-free survival of the patients, and only about one-third of the patients developed anti-GD3 antibodies (52). This subgroup also did not demonstrate significant survival benefits. The main reason for the failure may lies in the insufficient immunogenicity of the vaccine, which failed to induce an antibody response of sufficient intensity and proportion. Meanwhile, the expression of GD3 antigen in tumors is heterogeneous (only about 60% of patients have high expression), and it is distributed in normal tissues, resulting in low targeting efficiency and potential off-target toxicity risks (52).

### 3.4 Viral vector vaccine

Influenza A virus (IAV) modification is a promising strategy for lung cancer treatment (54). Ji, D. et al. designed a chimeric antigenic peptide influenza virus (CAP-Flu) system for delivering antigen peptides bound to influenza A virus (IAV) to the lung (55). The principle is to combine the attenuated IAV with the innate immunostimulant CpG. After intranasal administration to the lung of mouse, observed increased immune cell infiltration. Antigen ovalbumin (OVA) was then covalently displayed on IAV-CPG using click chemistry, and compared with the use of antigen peptides alone, this chimera caused a strong uptake of antigens by dendritic cells, a specific immune cell response, and a significant increase in tumor-infiltrating lymphocytes. They also engineered IAV to express anti-PD1-L1 nanobodies, which further enhanced the regression of lung metastases and prolonged the survival rate of mice after re-attack. This study suggests that engineered IAVs can be equipped with any tumor neoantigen to generate lung cancer vaccines and reveal the potential of viral vaccine vectors. TG4010 is a therapeutic cancer vaccine based on a modified vaccinia Ankara strain (MVA) encoding for the full-length cancer antigen Mucin 1 (MUC1) and human IL-2. The TIME trial is a placebo-controlled randomized phase II study aimed at evaluating the efficacy of TG4010 in chemotherapy for NSCLC. The results support that TG4010 treatment is associated with patient-specific T-cell response and prolonged overall survival (56).

The types of tumor vaccines in the field of lung cancer treatment and the main research are summarized in **Table 2**.

**Table 2:** The main types of vaccines in the field of lung cancer (classified by delivery platform).

Vaccine platform type	Subtypes	Core mechanism/ antigen source	Research team/reference	Research type/subject	
Cell vaccine	Tumor cell vaccine	GMCSF gene transduced autologous tumor vaccine (GVAX)	Kavita Maung (33, 34)	Clinical research: NSCLC
		Based on an adenocarcinoma line (AD100) transfected with B7.1 (CD80) and HLA A1 or A2.	Luis E Raez (35)	Clinical research: SCLC
	Autologous DC vaccine	Personalized neoantigen peptide pulsed autologous DC vaccine (Neo-DCVac)	Ding, Z. et al. (17)	Clinical research: (NCT02956551) 12 cases of treated metastatic lung cancer.
		Patient-derived organoid or cancer cell lysate-pulsed cationic nanoparticles (cNPs) to load immunogenic DC-derived microvesicles	Lihuan Shang. et al. (36)	Preclinical: in orthotopic pancreatic and lung cancer models
		Gene-modified bone marrow-derived DCs that secrete CCL21(CCL21-DC vaccine)	Ramin Salehi-Rad. et al. (37)	Preclinical: in murine NSCLC models.
		CCL21-DC vaccine	Michael S Oh. et al. (38)	Phase I clinical: For patients with resected NSCLC (6 cases)
	Allogeneic DC	Allogeneic plasmacytoid DC line (PDC*line)	Hannani, D. et al. (39)	Clinical: among lung cancer patients
	DC vaccine combined with ACT	The DC vaccine generates cells NRT	LI, Q. etc. (40)	Preclinical: in the pre-immunized mouse model of lung cancer
Nucleic acid vaccine	mRNA vaccine	mPLA/mRNA tumor vaccine (mLPR) encoding neoantigens	Shijie Ma. et al (18).	Preclinical
		mRNA encoding 7 different neoantigens	Nguyen, c.m. et al. (41)	Preclinical: in the LLC homologous mouse model
		BNT116 is an mRNA vaccine against co-tumor-associated antigens in NSCLC	Atmaca, Akin (42)	Clinical research: NSCLC
		mRNA-4157 (V940) is an mRNA vaccine targeting personalized neoantigens	Gainor, J. F (43)	Clinical research: NSCLC
Peptide/protein vaccine	Peptide vaccine	ALK vaccine	Ines Mota. et al. (47)	Preclinical: in HLA-transgenic mice
		Universal Cancer Peptide Vaccine (UCPVax)	Olivier Adotévi. et al. (48)	Phase Ib/IIa trial (NCT02818426): for treated advanced NSCLC.
	Protein vaccine	Bec2 is an anti-idiotypic antibody that mimics GD3, a ganglioside antigen	Giuseppe Giaccone (52)	Clinical research: SCLC
		CIMAvax-EGF vaccine is a recombinant human epidermal growth factor protein	Rodriguez, P. C (49–51).	Clinical research: NSCLC
Viral vector vaccine	Engineered influenza virus vector	combine the attenuated IAV with the innate immunostimulant CpG	Ji, D. et al. (55)	Preclinical: Lung cancer mouse model, intranasal administration.
		TG4010 is a therapeutic viral vaccine encoding human Mucin 1 and interleukin-2	Tosch, C (56).	Clinical research: NSCLC

GMCSF, Granulocyte Macrophage Colony-Stimulating Factor; DC, Dendritic cells; ACT, Adoptive cell therapy; NRT, Neoantigen-reactive T cells; ORR, Objective Response Rate; DCR, Disease Control rat; PFS, Progression-free survival; OS, Overall survival; NSCLC, Non-small cell lung cancer; IAV, Influenza A virus.

## 4 Breakthroughs in lung cancer tumor vaccines

In recent years, with the advancement of technologies such as informatics and engineering. Many breakthroughs have been made in the research of tumor vaccines, highlighting their great potential in treating lung cancer.

### 4.1 More effectively screen for new antigens

Most neoantigens are individually specific, and advancements in sequencing and bioinformatics technologies have made it possible to rapidly identify candidate neoantigens in individual patients (57). Currently, the most widely used approach involves exome sequencing (DNA sequencing) of tumor cells to detect mutations and identify neoantigens. New research indicates that a significant portion of neoantigens are caused by post-translational error splicing events and degradation and transport processes. The translational group holds significant potential in mining variant information and guiding individualized neoantigen identification (58). Lian, F et al. used high-throughput sequencing and bioinformatics analysis. Guided by the abundance of tumor mutations and the affinity of MHC peptides, individualized T cell epitopes were customized, thereby reducing the pool of candidate antigen peptides (58). Subsequently, they identified and prepared a neoantigen vaccine for lung cancer. The *in vitro* interferon -γ enzyme-linked immunospot assay proved that the neoantigen derived from the translation group has strong immunogenicity. Furthermore, the C57BL/6 mouse model based on subcutaneous tumors of lung cancer verified the potent anti-tumor effect of the neoantigen vaccine derived from the translation group. The immunofluorescence results confirmed the enhanced infiltration of T cells in the tumor tissue. Translation analysis sequencing has great potential in mining mutation information and guiding individualized neoantigen identification, and can select personalized neoantigens in an effective and feasible way.

### 4.2 Innovation in delivery platform technology

Researchers are constantly exploring better antigen delivery platforms. Among them, dendritic cell-derived exosomes (DEX) demonstrate unique potential as a tumor vaccine platform. Exosomes are nanomembrane vesicles released by various cells and play a crucial role in the movement of substances and information between cells (59). Research suggests that by loading TAAs or neoantigens onto exosomes (especially those derived from dendritic cells or tumor cells), they could efficiently activate antigen-specific CD8^+^ T cell and CD4^+^ T cell responses and induce long-term immune memory (60). A preclinical study successfully screened out neoantigens using patient-derived organoids and loaded them onto exosomes, and this personalized vaccine completely cleared the established tumors in a mouse model and produced memory protection (61). This indicates the great potential of personalized neoantigen exosome vaccines. The study of Shinji Morisaki et al. is likely to provide direct evidence for a lung cancer neoantigen exosome vaccine (62). They extracted DEX from the culture supernatants of dendritic cells of seven patients with advanced solid tumors who were treated with “neoantigen pulsed dendritic cell vaccine”. Monocytes and T lymphocytes were isolated from the peripheral blood of patients for research. The results showed that DEX derived from dendritic cells could transform quiescent monocytes into functional dendritic cells capable of presenting neoantigens. These “armed” monocytes can effectively activate the specific anti-cancer T cells in the patient’s body (62). This study verified the DEX function that had previously been mainly observed in animal models in human patient samples, providing a basis for clinical translation. The role of dendritic cell vaccines may not only be the direct activation of T cells by the reinfused dendritic cells, but also the DEX released by them can act as a “messenger” to convey antigen information to other immune cells such as monocytes in the body, forming a “relay” type of immune amplification effect (62). It can effectively address the potential risks of insufficient antigen delivery, weak lymph node homing and live cell transfusion that may exist in traditional DC vaccines (63).

### 4.3 The development of new adjuvants

Tumor vaccine adjuvants play a critical role in enhancing the immunogenicity of vaccines and improving the body’s immune response to tumor antigens. Researchers are actively investigating novel adjuvants to optimize antigen delivery processes. To date, several adjuvants have been successfully applied in preclinical lung cancer models. Acid-ionized iron nano-adjuvants offer a scalable and easily convertible strategy to enhance STING cascade activation and antigen cross-presentation, enabling personalized cancer vaccination immunotherapy (64). Monophophoric lipid A (mPLA) is a novel adjuvant and a toll like receptor 4 (TLR4) agonist. It can be inserted into the hydrophobic layer of liposomes due to its unique lipid properties, which escorted mRNA into the cytoplasm and improved the immune response (18). mPLA can induce local macrophage polarization through pattern recognition receptors at lower concentrations and maintain good biosafety compared with adjuvants such as cytokines and bacterial toxins. Liposome is vesicles composed of phospholipid bilayer, which can encapsulate antigens and adjuvants, thereby enhances antigen stability, promotes APC uptake, and can simultaneously induce humoral immunity and cellular immunity (65). Nanoparticles can precisely regulate the antigen release rate, target immune cells through surface modification, and enhance the adjuvant effect (66). Lipid nanoparticles, nanoemulsion, microvesicles and extracellular vesicles have been proven to be effective delivery adjuvants (36, 67–70).

### 4.4 New administration methods

Traditional types of cancer vaccines are mainly administered by intravenous injection (71). Li H et al. developed a non-invasive intranasal cancer vaccine utilizing circular RNA encapsulated in lipid nanoparticles to induce localized mucosal immune responses. In contrast, non-invasive respiratory mucosal delivery methods, such as intranasal or inhalation routes, directly stimulate the immune response at the respiratory mucosal site without inducing a systemic immune response. This strategy elicited potent anti-tumor T cell responses in preclinical lung cancer models while mitigating the systemic adverse effects commonly associated with intravenous RNA vaccination. Study indicated that the intranasal circRNA vaccine elicits a potent anti-tumor response with fewer side effects compared to other routes of administration (71).

Haoran Xu. et al. found that, compared with other vaccination regimens, the primary immune-booster vaccination method initiated with intramuscular DNA vaccines, followed by the intranasal attenuated live influenza vector vaccine (LAIV) booster, induced a higher frequency of lung CD8+ TRM cells (72). This strategy shapes the mucosal microenvironment and reprograms central memory T cells to generate lung-resident memory T cells that prevent lung metastasis, which can be considered for optimizing vaccine strategies.

## 5 The combined application strategy tumor vaccines in lung cancer

The immune escape of tumor cells is a key issue hindering the efficacy of tumor vaccines. The efficacy of a single treatment is limited, while combination therapy can enhance the therapeutic effect and prolong the response period through synergistic effects. The combination of tumor vaccines with other therapies, such as immune checkpoint inhibitors, radiotherapy and chemotherapy, and targeted therapy, shows great potential for development.

### 5.1 Combined with immune checkpoint inhibitors

Neoantigen-specific T cells can effectively regress tumors and prevent tumor recurrence or metastasis through memory T cells. However, the immune response it triggers can be negatively regulated by immune checkpoints, which may lead to T cell exhaustion and reduce the efficacy of anti-tumor immunity. Therefore, in order to enhance the effectiveness of cancer immunotherapy, it is beneficial to combine the role of neoantigens in triggering T-cell responses with immune checkpoint blockade (ICB) (73). “Cold” tumors based on lack of T cell infiltration show reduced potential for checkpoint inhibitor (CPI) therapy. Cancer vaccines may induce the needed antitumor T cell response to synergize with CPIs and overcome resistance (74). William Becker had effectively transition a (CPI)-resistant tumor into a CPI-susceptible tumor through the use of cancer vaccines in mice (74).

Tumor vaccines activate CD8+ cytotoxic T lymphocytes (CTLs) and CD4+ helper T cells by presenting TAAs or neoantigens, forming a “promotion-release inhibition” synergistic pathway with ICIs, which has become a key direction for breaking through drug resistance. Early clinical studies have confirmed that personalized neoantigen vaccine Neo-PV-01 plus nivolumab is feasible and safe, stimulating durable neoantigen-specific T cell reactivity in solid tumors (75).

Hannani, D. et al. used plasmacytoid dendritic cell line (PDC*line) as the antigen presentation platform and confirmed in lung cancer patients that anti-PD-1 therapy combined with tumor vaccine could synergistically enhance the activation of circulating anti-tumor CD8+ T cells, providing a basis for the clinical application of PDCline vaccine combined with ICI (39).

### 5.2 Dual immune compound vaccine: multi-target synergistic enhancement of immune response

The dual immune compound vaccine breaks through the limitations of a single target by integrating antigen delivery and immune regulation functions. The team of Guixue Yang studied and constructed the PEI lipid nanoparticles (PEI-LNP)/siRNA complex (EPV-PEI-LNP-SiRNA) with the therapeutic function of PD-L1-siRNA and the dual immune enhancement function of EGFR short peptide/PD-L1 (68). Used for the prevention and treatment of EGFR-positive lung cancer. Lipid nanoparticles deliver siRNA and EGFR short peptide vaccines to non-small cell lung cancer (NSCLC), increasing tumor invasion and the activation of CD8 + T cells. This compound vaccine achieves tumor-specific expression of immune-stimulating cytokines. The research results suggested that combined therapy is superior to single-target therapy.

Wuyi Zeng et al. generated specific Mesothelin (MSLN) fragment combined with PD-L1 and granulocyte-macrophage colony-stimulating factor (GM-CSF) peptide immunogen (MSLN-PDL1-GMCSF) (76). It was found that in the mice Lewis lung carcinoma models, DCs carrying the MSLN-PDL1-GMCSF vaccine triggered stronger endogenous anti-PD-L1 antibody and T-cell responses and antigen-specific cytotoxic T lymphocyte (CTLs) had cytolytic activity against tumor cells expressing both MSLN and PD-L1 simultaneously. This study demonstrated that inoculation with MSLN-PDL1-GMCSF effectively inhibited the tumor growth of MSLN+ and PD-L1+ lung cancer cells, showing significant therapeutic anti-tumor potential. Furthermore, PD-1 blockade further enhanced the synergistic anti-tumor therapeutic effect of the MSLN-PDL1-GMCSF vaccine in immunized mice. This MSLN therapeutic vaccine containing PD-L1 can induce sustained anti-PD-L1 antibody and CTL responses. By combining the MSLN-PD-L1-GMCSF vaccine and PD-1 blockers, it provides an effective immunotherapy strategy for lung cancer immunotherapy.

### 5.3 Combined with chemotherapy

Chemotherapy induces immunogenic death (ICD) of tumor cells to release antigens, forming a synergistic effect of “boosting immunity + increasing antigens” with vaccines. The phase 1b clinical trial conducted by Awad et al. showed that the personalized vaccine NEO-PV-01 combined with chemotherapy (carboplatin + pemetrexed) and pembrolizumab in the treatment of first-line metastatic non-squamous NSCLC significantly prolonged the progression-free survival (PFS) of patients and had good safety. Confirm the clinical feasibility of the combination of vaccines and chemotherapy (22).

R Zhong et al. conducted a prospective, single-arm Phase II clinical trial to investigate the safety and efficacy of lung cancer dendritic cell vaccine (DCVAC/LuCa) combined with standard carboplatin/pemetrexed in the treatment of advanced non-squamous (nsq) non-small cell lung cancer (NSCLC). The enrolled patients were stage IV nsq NSCLC without tumorigenic drivers and who had not received previous treatment. The treatment included carboplatin/pemetrexed for up to 6 cycles, followed by pemetrexed for 21 cycles or until progression or intolerance occurred. Patients who did not progress after two chemotherapy cycles began to receive subcutaneous DCVAC/LuCa (s.c) on the 15th day of the third cycle, and then on the 3rd week (the 15th day of the chemotherapy cycle), with a maximum of 15 doses. A total of 61 patients were included in this trial. Among the safe population (n = 60), 8 patients (13.33%) presented with grade 3 or above treatment associated adverse events (AEs), and 6 patients (10.0%) presented with serious AEs not related to leukocyte isolation or DC vaccination. Six cases of grade 1 AE were considered to be related to leukocyte segregation. No AEs induced by DCVAC/LuCa was observed. The 2-year survival rate of the modified intention-to-treat population (n = 44) was 52.57%. The median PFS was 8.0 months, and the ORR was 31.82%. It indicates that chemotherapy combined with vaccines can enhance the survival benefits of patients with advanced NSCLC.

### 5.4 Combined with targeted therapy

There have been attempts to combine “targeted + immunotherapy” in the field of lung cancer. Mechanistically, targeted drugs (such as EGFR-TKIs) can effectively induce immunogenic cell death (ICD) by directly killing cancer cells carrying specific driver genes (such as EGFR mutations) (77). The ICD induced by targeted therapy enhances the uptake of cancer cells and antigen presentation by antigen-presenting cells, which helps to activate anti-tumor immunity. Many targeted therapy drugs can directly or indirectly regulate the function of immune cells. For instance, anti-angiogenic drugs (such as bevacizumab) can improve tumor vascular function, promote T-cell infiltration into tumors, and reduce immunosuppression (78, 79). Additionally, drugs targeting certain pathways, such as CDK4/6 or PI3K inhibitors, can upregulate the expression of MHC in cancer cells, making them more easily detectable by the immune system (enhancing antigen presentation) (80, 81). Currently, there have been numerous reports on the combination of targeted drugs and immune checkpoint inhibitors. The IMpower150 (NCT02366143) and ORIENT-31 (NCT03802240) studies have shown significant benefits in both PFS and OS, demonstrating the synergistic effect of targeted therapy combined with ICI treatment (82–84).

Neoantigen vaccines have good safety profiles. For lung cancer subgroups defined by specific driver genes (such as EGFR, ALK, KRAS G12C), using corresponding targeted drugs in combination with personalized neoantigen vaccines or shared neoantigen vaccines targeting the same mutant antigens theoretically can achieve the most precise “targeted clearance + immune memory establishment”, which is expected to prolong treatment efficacy and overcome drug resistance (85, 86).

Currently, the formal clinical combination of “tumor vaccines + targeted drugs” in the field of lung cancer is still in the early stage of exploration. The EPICAL trial (NCT03623750) is a phase I/II study evaluating the safety and preliminary efficacy of the combination of tumor vaccine CIMAvax-EGF and osimertinib (a third-generation EGFR-TKI) in the treatment of patients with advanced NSCLC with EGFR mutations: This trial confirmed that even with potent TKIs (osimertinib), tumors may still rely on exogenous EGF signals (paracrine/autocrine). Depriving EGF can enhance the efficacy of TKIs and delay the emergence of drug-resistant clones (87).

Lin et al. evaluated a new antitumor strategy by adding neoantigen vaccine to the regimen of Bev and anti-PD-1 antibody (21). They confirmed in the orthotopic lung cancer model that the neoantigen vaccine combined with Bev and anti-PD-1 antibodies could significantly reduce tumor nodules and induce a high proportion of Ki67+ CD8+ T cell infiltration, suggesting that anti-angiogenic therapy creates a favorable immune microenvironment for the vaccine (21).

## 6 Characteristics of clinical trials vaccines in lung cancer

A comprehensive analysis of the current landscape of clinical trials is instrumental in gaining insights into the application status and the most recent advancements of tumor vaccines in the treatment of lung cancer. A study conducted a descriptive study of clinical trials for NSCLC therapeutic vaccines registered on ClinicalTrials.gov through March 17, 2022 (46). They encompassed 117 registered trials, among vaccine types, protein/peptide vaccines account for the highest proportion (41.88%), followed by dendritic cell vaccines (18.80%) and tumor cell vaccines (14.53%). The main characteristic of these trails is lack of randomized control, lack of mask, and recruiting less than 50 participants (46).

We retrieved the lung cancer therapeutic vaccine clinical trial data from ClinicalTrials. gov database (https://clinicaltrials.gov) to. Set the search parameter to the disease: lung cancer; Intervention/treatment: Vaccine. As of June 30, 2025, a total of 277 relevant clinical trials have been identified (**Supplementary Document 1**). Among these trials, 128 (46.2%) were marked as completed. Other states include before recruiting (22, 7.9%), recruiting (37, 13.3%), withdrawn (17, 6.1%), terminated (31, 11.2%), suspended (3, 1.1%) and unknown (39, 14.1%). In terms of vaccine types, there are, mix vaccine (MV), dendritic cell vaccines, protein/peptide vaccines, DNA vaccines, mRNA vaccines, viral vector vaccines and tumor cell vaccines. The combined treatment strategy includes the combination of PD1/PDL1, chemotherapy. Phase 1 and Phase 2 clinical trials are more frequent, which indicated that the research on lung cancer vaccines is still in the exploratory stage, mainly focusing on the preliminary verification of efficacy and safety. From 2022 to 2025, 45 projects were initiated, showing a growth trend compared with previous periods. By comparing previous study, it is not difficult to see that in recent years, the number of trials of therapeutic vaccines for lung cancer has been continuously increasing, the types of vaccines have become more diverse (mRNA vaccines, dc vaccines), and combination therapy has been widely attempted. The types of experiments are still mainly early-stage.

In summary, the clinical trials of lung cancer vaccines are currently in a multi - phase exploratory stage. Future research should be more precise in targeting different lung cancer subtypes, delving deeper into individualized and combinatorial treatment strategies for vaccines. This approach is expected to enhance the overall treatment efficacy for lung cancer and ultimately improve the quality of life for patients.

## 7 Summary and prospect

The development history of lung cancer vaccines has evolved from extensive exploration to precise focus. Early genetically engineered vaccines represented by GVAX have enhanced immunogenicity through genetic engineering (transfected with GM-CSF), confirming that they can induce specific T-cell responses even in “cold tumors” (34). B7.1/HLA-modified allogeneic tumor cell vaccines have demonstrated the feasibility of breaking immune tolerance by providing key co-stimulatory signals (35). However, as single therapies, none of them have demonstrated efficacy sufficient to change clinical practice in advanced lung cancer. The main inspirations from the above two tumor cell vaccines for us lie in: Although the whole-cell antigen spectrum is broad, it is difficult to overcome the powerful tumor immunosuppressive microenvironment. Combined therapy helps to improve this deficiency, suggesting the necessity of combined therapy. Anti-idiotypic antibody vaccines represented by Bec2/BCG attempted to target glycolipid antigens such as GD3. The result did not demonstrate significant survival benefits in the Phase III clinical trials of SCLC (52), It highlights the limitations of a single antigen: the target expression is heterogeneous, and it mainly induces humoral immunity, making it difficult to generate a strong anti-tumor T-cell response. Therefore, although early exploration did not lead to widely successful therapies, it laid an indispensable scientific and clinical foundation for the second-generation precision vaccines.

Therapeutic vaccines represented by CIMAvax-EGF, BNT116, mRNA-4157 have provided us with valuable experience. It laid the foundation for subsequent research on individualized tumor vaccines and combined immunotherapy. These experiences have directly driven the evolution of current vaccine strategies: from whole-cell vaccines to precisely targeted neoantigen mRNA or peptide/protein vaccines; From relying on a single antigen to developing multivalent vaccines; From the sole use of vaccines to emphasizing combined treatment models with immune checkpoint inhibitors, chemotherapy, radiotherapy, etc. Therefore, the current frontier of lung cancer vaccines has shifted to mRNA/peptide vaccines based on individualized neoantigens, and is bound to form a combined strategy with therapies such as immune checkpoint inhibitors. In addition, significant progress has also been made in the research of dc cells themselves or exosomes derived from them as delivery platforms, which has made DC-based vaccines one of the hotspots in tumor vaccine research.

In summary, advancements in sequencing and bioinformatics technologies have facilitated the rapid identification of candidate neoantigens for individual patients via sophisticated, customized algorithms. The clinical development of lung cancer vaccines has evolved from traditional antigen delivery systems to an era characterized by precise personalization. Breakthroughs in DC vaccines (such as exosome vaccine-derived from DC), mRNA technology, adjuvant optimization, vaccine administration routes, and overall vaccine design have enabled researchers to develop and validate more precise and potent lung cancer vaccines. This progress has expedited the translation of lung cancer vaccines from laboratory research into broad clinical application. Overall, therapeutic cancer vaccines have continuously demonstrated their therapeutic potential by targeting neoantigens, combining with immune checkpoint inhibitors, and integrating with targeted therapies, and are expected to become a regular guest in the comprehensive treatment of lung cancer.
